# Efficacy and Safety of Cuban Policosanol on Cognitive Function and Antioxidant Status in Adults with Subjective Memory Decline: A 12-Week Randomized, Double-Blinded, Placebo-Controlled Multicenter Trial

**DOI:** 10.3390/nu18213496

**Published:** 2026-10-24

**Authors:** Kyung-Hyun Cho, Yeong-Bae Lee, Min Ju Kang, Dhananjay Yadav

**Affiliations:** 1 Raydel HDL Research Institute, Medical Innovation Complex, Daegu 41061, Republic of Korea; yadav11@raydel.co.kr; 2 Department of Neurology, Gil Medical Center, Gachon University College of Medicine, Incheon 21565, Republic of Korea; yeongbaelee@gachon.ac.kr; 3 Department of Neurology, Veterans Health Service Medical Center, Seoul 05368, Republic of Korea; minju.kang@bohun.or.kr

**Keywords:** policosanol, subjective memory decline, cognitive function, ADAS-Cog, superoxide dismutase (SOD), K-MMSE

## Abstract

**Objectives**: Early non-pharmacological interventions for people with subjective memory decline (SMD) are increasingly advocated in preventing age-related cognitive decline and potentially delaying progression to mild cognitive impairment or dementia. In this multicenter clinical trial (RAYDELP1), we evaluated the efficacy and safety of sugarcane-derived Cuban policosanol (PCO) on cognitive function, superoxide dismutase (SOD) activity, inflammatory markers, and metabolic panels in adults aged 55–85 years with subjective cognitive complaints. **Methods**: A 12-week, randomized, double-blinded, placebo-controlled trial was conducted at Gachon University Gil Medical Center and Seoul Veterans Hospital in adults with SMD complaints. Individuals were randomized equally into three groups: placebo, PCO 20 mg group, or PCO 40 mg group. Assessment of cognitive function was performed using the Alzheimer’s Disease Assessment Scale–Cognitive Subscale (ADAS-Cog 11) and the Korean Mini-Mental State Examination (K-MMSE). All measurements, including SOD activity, inflammatory markers, and metabolic parameters, were analyzed in blood samples at baseline and after 12 weeks. For an exploratory subgroup analysis, participants receiving statins and those with potential ceiling effects or greater cognitive impairment based on K-MMSE scores were excluded. The K-MMSE range of 18–26 was used as an exploratory criterion to reduce heterogeneity in baseline cognitive scores, resulting in a refined analysis population of 56 participants. **Results:** In the refined SMD population, SOD activity increased significantly in the PCO 40 mg group after 12 weeks (mean change, +0.16 ± 0.25 U/mL; *p* = 0.0109) with increasing HDL-C, whereas changes in the placebo groups were not statistically significant (+0.08 ± 0.24 U/mL, *p* = 0.1873) with decreasing HDL-C. HDL-C levels increased in a dose-dependent manner in both the PCO 20 mg (+0.78 ± 5.1 mg/dL) and PCO 40 mg (+2.2 ± 6.7 mg/dL) groups within and between the groups. Both PCO groups showed significant differences compared with placebo in the between-group analysis and baseline-adjusted ANCOVA (*p* = 0.0124 and *p* = 0.0142, respectively). K-MMSE place-orientation scores improved significantly from baseline within both PCO groups (20 mg: 0.94 ± 0.87, *p* = 0.0003; 40 mg: 0.47 ± 0.77, *p* = 0.0155), whereas no significant within-group change was observed in the placebo group. Similarly, ADAS-Cog 11 language scores improved significantly within both PCO groups (20 mg: −1.17 ± 1.92, *p* = 0.0194; 40 mg: −0.95 ± 1.58, *p* = 0.0176). The 20 mg PCO group also showed a significant within-group decrease in Visual CPT false-alarm errors (−3.83 ± 7.13, *p* = 0.0357). However, between-group comparisons and baseline-adjusted ANCOVA did not demonstrate statistically significant differences in the selected cognitive outcomes. **Conclusions:** In the analyzed study population, HDL-C levels increased numerically in both PCO groups in a dose-dependent manner. The PCO 40 mg group showed a significant within-group increase in serum SOD activity, while improvements in selected cognitive domains were observed within both 20 mg and 40 mg PCO groups. However, no significant differences were found in between-group or ANCOVA analyses. PCO was well tolerated, and further clinical studies are warranted to confirm these exploratory findings and clarify its potential effects on cognitive function and enhancement of HDL quantity and quality.

## 1. Introduction

Aging is a global challenge that cannot be stopped, but healthy lifestyle measures and early interventions may slow its progression [1,2]. Regular monitoring by public health professionals, researchers, clinicians, and policymakers can help assess the epidemiological and demographic burden of aging across regions and support the development of appropriate strategies [3]. Aging is also associated with various health conditions, including cardiovascular diseases and neurological dysfunction, which are increasingly prevalent among older adults [4,5,6]. Recent studies report that older adults may experience changes in memory, attention, and recognition, which can affect daily activities and overall quality of life [7,8].

During the course of cognitive aging, some individuals report memory difficulties although performing within the normal range on objective neuropsychological assessments and not meeting the diagnostic criteria for mild cognitive impairment (MCI) or dementia [9,10,11]. These individuals are commonly described as having subjective memory decline (SMD). While individuals with SMD have a significantly increased risk of progressing to MCI and Alzheimer’s disease (AD) [12,13], there is a need for safe, long-term, non-pharmacological interventions using bioactive agents that may help preserve cognitive health and potentially delay cognitive decline and dementia [14,15].

Cuban policosanol (PCO), sugarcane wax alcohol, is an alternative bioactive agent that has been extensively investigated for its potential effects on blood pressure and lipid levels via improving HDL quantity and quality in both in vitro and in vivo models [16,17,18]. Recent clinical trials have also suggested potential protective effects of PCO against various health conditions [19,20,21,22]. Policosanol is a purified mixture of high-molecular-weight aliphatic fatty alcohols, primarily containing 24–34 carbon atoms, extracted from the wax of *Saccharum officinarum* L. It has been observed that policosanol may have potent neuroprotective and cognitive effects through its antioxidant, anti-inflammatory, glycation-reducing, and HDL-modulating properties [19,23,24]. In a recent 30-week zebrafish model of high-cholesterol/high-galactose-induced metabolic stress, PCO reduced brain reactive oxygen species (ROS) production and cellular senescence and improved impaired swimming behavior, suggesting a potential beneficial effect on cognitive function. Additionally, the PCO supplementation enhanced swimming distance by approximately 5-fold compared with the metabolically stressed group. This study also reported reduced brain 4-hydroxynonenal (4-HNE) accumulation and oxidative/apoptotic damage, suggesting a potential role for PCO in protecting neuronal integrity [25]. Our group’s previous work in an in vitro model demonstrated that PCO incorporated into reconstituted HDL enhanced viability and proliferation while reducing apoptosis and ROS production in BV-2 microglial cells, suggesting that improved HDL functionality could contribute to neuroprotective effects [16]. Preclinical evidence suggests that PCO may reduce Aβ plaque accumulation and activation of microglial cells in 5xFAD mice, accompanied by lowering pro-inflammatory cytokines, including IL-6 and TNF-α, which may reduce neuroinflammatory stress and improve memory performance [15]. PCO improved spatial learning and memory, potentially by reducing hippocampal long-term potentiation (LTP) impairment, modulating oxidative stress, and suppressing Aβ plaque formation [26]. Importantly, clinical studies have reported some neurological effects of PCO, although evidence for a direct effect on memory remains limited. Fontani et al. reported in a double-blind, placebo-controlled study of 30 healthy young adults that 7 days of isopolicosanol and octacosanol supplementation (3.6 mg/day) [provided by ALSO Laboratories, Milan, Italy] reduced reaction time and altered event-related potentials, including increased contingent negative variation and shorter P300 latency in both aforementioned groups [27]. A randomized study in 18 karateka athletes found that 21 days of omega-3 fatty acids combined with PCO (10 mg/day) reduced reaction time and improved vigor [28]. The reduction in reaction time was accompanied by earlier movement-related brain potentials and electromyography (EMG) activation, suggesting an effect on central nervous system responses. However, researchers could not determine the individual effect of PCO because it was administered with omega-3 fatty acids [28]. In an observational study of 50 patients with ischemic stroke, PCO (20 mg/day) was given with antiplatelet therapy and vitamins for 5 years. Neurological scores, assessed with the modified Canadian Neurological Scale, improved during follow-up, and PCO showed a favorable safety profile. The reduction in total cholesterol was strongly associated with neurological improvement. Only one patient experienced a recurrent stroke, suggesting a possible benefit of PCO in neurological recovery and vascular outcomes [29]. A clinical analysis evaluated the effects of PCO (20 mg/day) plus aspirin in 142 hypertensive patients with recent non-cardioembolic ischemic stroke. Patients receiving PCO plus aspirin showed a significant reduction in modified Rankin Scale (mRS) scores during the 6-month treatment period. At the end of treatment, 80.3% of patients in the PCO group achieved an mRS score ≤1, compared with 8.5% in the placebo plus aspirin group. These findings imply that PCO combined with aspirin may improve functional recovery after ischemic stroke [30]. Existing human studies of PCO have primarily evaluated reaction time, attention [27], electrophysiological responses, or neurological recovery [30] rather than standardized cognitive outcomes such as memory, Mini-Mental Status Examination (MMSE), Montreal Cognitive Assessment (MoCA), or Alzheimer’s Disease Assessment Scale–Cognitive Subscale (ADAS-Cog). Moreover, some studies involved combination treatments or lacked adequate placebo controls, making it difficult to establish a specific effect of PCO on cognition [28]. Despite these encouraging findings, the potential cognitive advantages of PCO in humans remain unclear. In particular, evidence from human clinical trials on the effects of PCO on memory and cognitive performance and their association with systemic antioxidants and inflammatory markers remains limited. To address these gaps, the present multicenter, randomized, double-blinded, placebo-controlled RAYDELP1 trial evaluated the effects of 20 and 40 mg/day of sugarcane wax-derived PCO for 12 weeks in 56 adults aged 55–85 years with subjective memory loss. Cognitive function was assessed using ADAS-Cog 11, K-MMSE, and Visual Continuous Performance Test (CPT), along with blood metabolic panels, antioxidant SOD, and pro-inflammatory cytokines.

## 2. Materials and Methods

### 2.1. Policosanol

The Cuban sugarcane wax alcohol (policosanol) was sourced from the National Centre for Scientific Research (CNIC), Havana, Cuba, and was complementarily provided by Raydel Pty Ltd., Thornleigh, NSW, Australia. The genuine policosanol consisted of a characteristic mixture of eight long-chain aliphatic alcohols (LCAAs): 1-tetracosanol (C24, 0.01–2.0%), 1-hexacosanol (C26, 3.0–10.0%), 1-heptacosanol (C27, 0.1–3.0%), 1-octacosanol (C28, 60–70%), 1-nonacosanol (C29, 0.1–2.0%), 1-triacontanol (C30, 10–15%), 1-dotriacontanol (C32, 5–10%), and 1-tetratriacontanol (C34, 0.1–5.0%) with ≥90% purity, as determined by the manufacturer’s validated gas chromatography (GC) method [31]. The active tablets contain PCO at 10 or 20 mg/tablet (batch number: 2024-146) (Supplementary Table S3) corresponding to daily doses of 20 or 40 mg, respectively. The dose-selection rationale was documented in the clinical study report. The doses selected for the RAYDELP1 trial were based on a previous animal experiment in which Cuban policosanol was administered orally at 5 mg/kg in 5xFAD mice, an experimental model of Alzheimer’s disease [15]. The human-equivalent dose (HED) was estimated using body-surface-area conversion, based on a mouse dose of 5 mg/kg, using the formula: HED (mg/day) = 5 mg/kg × (3/37) × 60 kg, yielding approximately 24.3 mg/day for a 60 kg adult. Accordingly, 20 mg/day was selected as the lower dose. Moreover, the Korean Ministry of Food and Drug Safety (MFDS; formerly KFDA) recognizes 20 mg/day of PCO derived from sugarcane wax alcohols as a daily intake level that may help regulate blood pressure and cholesterol levels in humans, providing a regulatory basis for the selection of this dose in the present study [32]. The higher dose of 40 mg/day was included to explore whether a greater daily intake might produce different effects on cognitive and biochemical outcomes. Previous work conducted by Ortiz et al. 2001 & Arruzazabala et al. 2002 investigated PCO at 40 mg/day for lipid-related and antiplatelet outcomes, though additional benefits over 20 mg/day were not consistently established [33,34]. The investigation and placebo products were designed to be identical in appearance, coating, disintegration characteristics, and organoleptic properties to maintain double blinding were manufactured by Cosmax Bio Co., Ltd. (Jecheon, Republic of Korea) (Supplementary Table S4). The placebo tablets were identical in appearance and formulation, with PCO replaced by an equivalent amount of lactose and crystallized cellulose.

### 2.2. Study Design and Participants

RAYDELP1 was a 12-week, multicenter, randomized, double-blinded, placebo-controlled, parallel-group human intervention study designed to evaluate the efficacy and safety of PCO in individuals (aged 55–85 years) reporting SMD. The trial was designed and supervised by NeoNutra Co., Ltd. (Seoul, Republic of Korea) as clinical research organization (CRO), and conducted at Gachon University Gil Medical Center and Seoul Veterans Hospital after obtaining official approval from the Institutional Review Board (IRB) of each clinical site. The study was approved by the Institutional Review Boards of Gachon University Gil Medical Center (IRB No. GB-2022-01) and Seoul Veterans Hospital (IRB No. BOHUN-2022-02-024). Clinical Trial Registration: The study was retrospectively registered with the Clinical Research Information Service (CRIS, Osong, Republic of Korea), under registration number KCT0012761. The registration application was submitted on 2 October 2026, and registration was completed on 11 October 2026. https://cris.nih.go.kr/cris/search/detailSearch.do?search_lang=E&focus=reset_12&search_page=L&pageSize=10&page=undefined&seq=35230&status=5&seq_group=35058 (date of accessed 19 October 2026).

The study compared baseline and follow-up cognitive measures, including different metabolic panels, antioxidant SOD, and inflammatory markers. Participants were randomized to receive policosanol at 20 mg, policosanol at 40 mg, or placebo for 12 weeks. Cognitive and other efficacy assessments were performed at baseline and during follow-up, including ADAS-Cog 11, Visual CPT, and K-MMSE.

### 2.3. Participant Disposition and Analysis Populations

**Figure 1:**
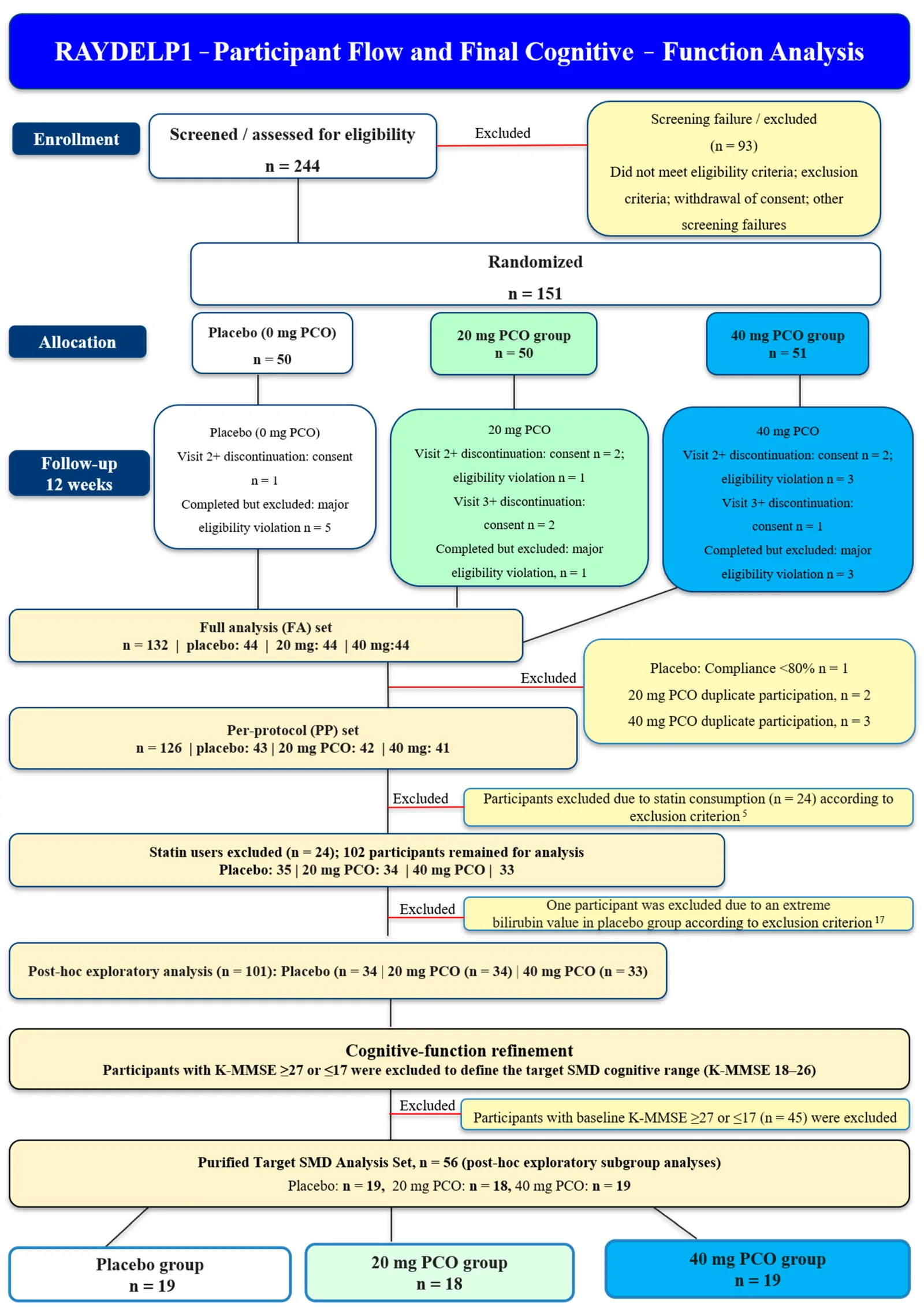
Overview of the RAYDELP1 study, selection and refinement of the exploratory cognitive analysis population based on post hoc K-MMSE criteria, resulting in a final analysis population of 56 participants. Criteria 5 and 17, as detailed in Supplementary Table S2, were applied for participant exclusion. PCO, policosanol; SMD, subjective memory decline; K-MMSE, Korean-mini mental state examination; FA set, Full analysis set; PP set, Per-protocol set.

A total of 244 individuals were screened, and 151 participants were randomized to the placebo (*n* = 50), 20 mg PCO (*n* = 50), or 40 mg PCO (*n* = 51) groups. Of these, 132 participants were included in the Full Analysis Set (FAS), with 44 participants in each treatment group. 19 participants were excluded from the FA Set because of withdrawal of consent, eligibility/ineligibility violations leading to discontinuation, or major eligibility/exclusion-criterion violations identified among participants who completed the study. Among the 132 participants in the FA Set, 6 were subsequently excluded from the Per-Protocol (PP) Set: (5 because of duplicate participation, 1 because of mean treatment compliance below 80%). The resulting PP Set consisted of 126 participants, including 43 in the placebo group, 42 participants in the 20 mg PCO group, and 41 in the 40 mg PCO group. For the focused cognitive-function analysis, participants receiving statin therapy (*n* = 24) were excluded from the PP population, resulting in a statin-excluded population of 102 participants. One participant in the placebo group was excluded from further analysis due to an extremely elevated bilirubin value, resulting in a total of 101 participants, as shown in Figure 1. This population was subsequently evaluated for baseline cognitive heterogeneity. The original protocol allowed enrollment on the basis of either a reduction in Seoul Neuropsychological Screening Battery-II (SNSB-II) verbal or visual memory performance of ≥1 SD relative to age- and education-matched norms or a K-MMSE score ≤26. The analysis report identified that this approach resulted in the inclusion of participants with a broad range of baseline cognitive performance. Participants with extreme baseline K-MMSE scores were excluded from the refined analysis: scores ≥27 were considered susceptible to a ceiling effect, whereas scores ≤17 represented cognitive impairment outside the intended target range. Participants with baseline K-MMSE scores of 18–26 were therefore retained as the target SMD population. The exclusions were intended to reduce heterogeneity in baseline cognitive performance and address potential ceiling effects among participants with higher K-MMSE scores, while limiting the inclusion of participants with lower scores who might have greater cognitive impairment. However, because K-MMSE scores alone cannot establish an SMD diagnosis or confirm the absence of objective cognitive impairment, this subgroup should be interpreted as an exploratory cognitive-analysis population rather than a definitively characterized SMD cohort.

The resulting exploratory analysis population comprised 56 participants: 19 in the placebo group, 18 in the 20 mg PCO group, and 19 in the 40 mg PCO group. The participant flow and successive analytical populations are summarized in Figure 1. This randomized, double-blind, placebo-controlled clinical trial is reported in accordance with the CONSORT 2025 guidelines. The completed CONSORT 2025 checklist is provided as a separate file, and the participant flow diagram is presented in Figure 1.

### 2.4. Exclusion Criteria of the Study

Participants were screened for exclusion criteria, such as clinically significant immune, respiratory, gastrointestinal/hepatobiliary, renal/urinary, or neurological disorders; use of medications or supplements that could affect cognitive function; substantial clinical issues related to thyroid, renal, hepatic, hypertension, or diabetes abnormalities; allergy or sensitivity to study-product ingredients; pregnancy or lactation; recent participation in any other clinical study; and additional conditions arbitrated by the investigator to make participation inappropriate. The detailed exclusion criteria are provided in Supplementary Table S2. The study product PCO was administered orally once daily for 12 weeks. Participants assigned to the 20 mg PCO group received two 10 mg PCO tablets at once (total dose, 20 mg), whereas participants in the 40 mg PCO group consumed two 20 mg PCO tablets (total dose, 40 mg). Participants in the placebo group consumed the placebo using the same dosing schedule.

### 2.5. Labeling and Allocation Blinding

To maintain accurate blinding, all products were packaged in identical high-density polyethylene (HDPE) bottles with no visible treatment identifiers. An independent operational team member, entirely isolated from the sponsor and clinical investigators, performed the labeling using pre-printed randomized codes (site-specific patient identification numbers). The randomization master key remained securely locked and inaccessible to the sponsor, clinical research organization (CRO) operations, and clinical sites until completion of the database lock, thereby sustaining treatment blinding throughout the study.

### 2.6. Efficacy Assessment and Cognitive Measures

Efficacy assessments were performed at baseline and after 12 weeks of supplementation, with the primary analysis focusing on changes from baseline to week 12. The study evaluated changes in metabolic parameters, inflammatory biomarkers, antioxidant SOD activity, and cognitive function, including the ADAS-Cog 11 language [35], K-MMSE temporal and place orientation, and Visual CPT false alarm errors from baseline to week 12 [36,37,38].

### 2.7. Anthropological and Laboratory Measurements

Blood samples were collected following an 8 h overnight fasting. Biochemical, metabolic, and inflammatory parameters were assessed to evaluate systemic safety and potential biological effects of PCO. Blood pressure (BP) measurements, including systolic BP (SBP) and diastolic BP (DBP), and BMI were assessed at baseline and after 12 weeks of PCO supplementation. Demographic characteristics, including age, sex, marital status, education level, and occupation, were assessed at Visit 1 using participant-reported information via self-questionnaire.

### 2.8. Serum Biochemical Parameters

Serum biochemical parameters included glucose, uric acid, albumin, total bilirubin, aspartate aminotransferase (AST), alanine aminotransferase (ALT), alkaline phosphatase (ALP), blood urea nitrogen (BUN), and creatinine. Serum lipid concentrations included total cholesterol (TC), triglycerides (TG), high-density lipoprotein cholesterol (HDL-C), and low-density lipoprotein cholesterol (LDL-C), TG/HDL-C, LDL/HDL-C, and HDL-C/TC (%). Biochemical analyses were performed using the Cobas c 502 chemistry analyzer (Roche Diagnostics GmbH, Mannheim, Germany).

### 2.9. Inflammatory and Antioxidant Biomarkers

Interleukin-6 (IL-6) and tumor necrosis factor-α (TNF-α) were measured using commercially available enzyme-linked immunosorbent assay (ELISA) kits according to the manufacturers’ instructions. The kits used were Human IL-6 Quantikine^®^ HS ELISA (R&D Systems, HS600C, Minneapolis, MN, USA) and Human TNF-α DuoSet^®^ (R&D Systems, DY210-05, Minneapolis, MN, USA). Superoxide dismutase (SOD) activity was measured using a commercial colorimetric SOD activity assay kit (Thermo Fisher Scientific/Invitrogen, EIASODC, Life Technologies Corporation, Frederick, MD, USA). These biomarkers were selected to characterize systemic inflammatory status. Serum SOD activity and serum concentrations of IL-6 and TNF-α were measured at the Department of Pharmacology, College of Medicine, Gachon University (Incheon, Republic of Korea), using the laboratory’s standard assay procedures. The measurements were performed under blinded conditions, and the raw measurement values were reported to the data center of the CRO, NeoNutra (Seoul, Republic of Korea). The laboratory was not involved in unblinding, statistical analysis, selection of the analysis population, or interpretation of the results.

### 2.10. Sample Size and Statistical Power Treatment

The sample size for the parent randomized controlled trial was determined prospectively based on the primary efficacy endpoint, change in ADAS-Cog 11 score, with 80% power and a two-sided significance level of 0.05. Allowing for an anticipated 25% dropout rate, approximately 50 participants per group were planned for enrollment (150 participants in total). For the present post hoc exploratory cognitive analysis, participants were selected from the completed study population using the refined analysis criteria, resulting in a final population of 56 participants. For the present secondary cognitive analysis, a refined SMD analysis set was derived from the study population based on baseline cognitive characteristics to reduce heterogeneity in baseline cognitive performance. This refined analysis included 56 participants (19 in the placebo group, 18 in the 20 mg PCO group, and 19 in the 40 mg PCO group) based on the baseline K-MMSE scores. This subgroup represented a refined analytical population from the completed trial rather than a separately recruited cohort.

### 2.11. Statistical Analysis

The SPSS software (version 29, Chicago, IL, USA) was used to determine statistical differences. Continuous variables are represented as mean ± standard deviation (SD), whereas categorical variables are represented as frequencies and percentages. All statistical tests were two-sided, and a significance level of *p* < 0.05 was used. For baseline demographic characteristics, continuous variables were compared among the three independent groups using one-way analysis of variance (ANOVA) or the Kruskal–Wallis test, depending on the data distribution. Categorical variables were compared using the chi-square test or Fisher’s exact test, as appropriate. For efficacy outcomes, within-group changes from baseline to week 12 were analyzed using the paired *t*-test. Between-group differences in change from baseline were assessed using one-way ANOVA or the Kruskal–Wallis test, as appropriate based on normality. To account for potential baseline differences, we performed an additional analysis of covariance (ANCOVA) using the baseline value of each efficacy variable as a covariate. No global adjustment for multiplicity across the multiple endpoints and comparisons was applied. Accordingly, the reported *p*-values should be interpreted as nominal, and statistically significant within-group findings should be considered exploratory. Between-group comparisons and baseline-adjusted ANCOVA were used to evaluate differences among treatment groups.

## 3. Results

### 3.1. Subject Distributions and Demographics

Baseline demographic characteristics are shown in Table 1. The placebo, 20 mg PCO, and 40 mg PCO groups were comparable with respect to age, sex, height, weight, BMI, and education level. Mean age was 61.84 ± 5.32 years in the placebo group, 63.11 ± 6.94 years in the 20 mg PCO group, and 63.53 ± 7.76 years in the 40 mg PCO group, with a *p*-value of 0.8769. The placebo group had a narrower age range (55–73 years), whereas the 40 mg PCO group showed a wider range (55–81 years) and a numerically higher mean age. Mean BMI was also numerically lower in the 40 mg PCO group than in the placebo group. These observations suggest some baseline heterogeneity among the groups and may be considered when interpreting the PCO-related findings. The study did not observe significant differences in sex distribution (*p* = 0.7735), height (*p* = 0.8390), weight (*p* = 0.6350), or BMI (*p* = 0.4185). Education level plays a significant role in cognitive function, and reports suggest that higher levels of education are associated with better cognitive performance. We found that education level was similarly distributed among the placebo, 20 mg PCO, and 40 mg PCO groups (*p* = 0.7805). Most participants had attained high school education or higher, including college/associate-level or university education.

### 3.2. Metabolic, Inflammatory, and Cognitive Outcomes

This section describes changes in metabolic, inflammatory, and cognitive parameters measured in the placebo, 20 mg PCO, and 40 mg PCO groups at baseline and week 12.

#### 3.2.1. Metabolic, Inflammatory, and Cognitive Changes in the Placebo Group

The metabolic, inflammatory, and cognitive outcomes of participants receiving placebo at baseline and week 12 are presented in Table 2. In the placebo group, several metabolic and inflammatory parameters changed in an unfavorable direction over the 12 weeks, especially lipid profiles.

There was a reduction in SBP and DBP observed in the placebo group, which may partly reflect their relatively higher baseline blood pressure and regression toward the mean. Although the placebo group was numerically younger, age was equivalent among groups (*p* = 0.8769), and the BP outcome can be interpreted as a within-group change. HDL-C decreased by approximately 7.0%, from 54.3 ± 14.5 to 50.5 ± 15.6 mg/dL (*p* = 0.0245). Additionally, other lipid parameters, triglyceride concentrations increased slightly, accompanied by a 7.9% surge in the TG/HDL-C ratio and a 5.6% reduction in the HDL-C/TC ratio. However, these changes were not statistically significant. In addition, the LDL-C/HDL-C ratio increased by approximately 7.5%, from 2.1 ± 0.7 to 2.3 ± 0.7 (*p* = 0.0146), which showed a higher trend after 12 weeks of placebo intake in this group. Collectively, the placebo group showed a significant decrease in HDL-C and an increase in the LDL-C/HDL-C ratio, together with slight changes in other lipid ratios over the 12 weeks. In addition to this, the inflammatory cytokines IL-6 increased by approximately 7.4% and TNF-α by 3.1%, although the changes were not statistically significant (*p* = 0.7346 and *p* = 0.5860, respectively). Antioxidant SOD activity was slightly increased, but the change was not significant (*p* = 0.1873). Selected cognitive measures showed a slight upward trend in the placebo group; however, no significant between-group differences were observed. The reason for this trend was not clear.

#### 3.2.2. Metabolic, Inflammatory, and Cognitive Changes in the 20 mg PCO Group

Table 3 presents the outcomes of the different parameters measured at baseline and week 12 in the 20 mg PCO group. Within-group analyses using paired *t*-tests showed a favorable trend in the 20 mg PCO group. The participants in the 20 mg PCO group at baseline and at 12-week follow-up showed a reduction in SBP and DBP, while HDL-C showed a slight elevation although no significance was detected. The inflammatory markers and SOD activity were varied and a favorable increase was reported for the 20 mg PCO intake group compared with the placebo group at 12 weeks of follow-up.

Significant improvements were observed in ADAS-Cog 11 language scores (*p* = 0.0194), Visual CPT false-alarm errors (*p* = 0.0357), and K-MMSE place orientation (*p* = 0.0003) in 20 mg PCO consumption participants. These findings indicate favorable changes in language, attentional control, and orientation-related cognitive performance in this group after 12 weeks of PCO consumption. However, as these were within-group results, they should be interpreted as preliminary evidence and require a larger controlled study for validation.

#### 3.2.3. Metabolic, Inflammatory, and Cognitive Changes in the 40 mg PCO Group

Table 4 presents the outcomes of the various parameters measured at baseline and week 12 in the 40 mg PCO treatment group. Within-group analyses that resulted from the paired *t*-test showed a protective trend across the 40 mg PCO group. In the 40 mg PCO group, SBP, DBP, TC, TG, LDL-C, TG/HDL-C, and LDL/HDL-C were lowered in this group after comparing with their baseline values, although they did not reach significance. HDL-C also increased by approximately 3.7%, along with an increase in HDL-C/TC (%) that showed a positive trend in this group after intake of PCO for 12 weeks compared with the baseline. At week 12, TG and LDL-C levels decreased numerically by 9.66% and 3.39%, respectively, in the 40 mg PCO group compared with baseline. SOD activity increased significantly (*p* = 0.0109) by approximately 20%, suggesting enhanced antioxidant capacity, whereas IL-6 and TNF-α decreased by approximately 33% and 13%, respectively, although these changes did not reach statistical significance.

Cognitive performance also improved significantly, with reductions in ADAS-Cog language scores (*p* = 0.0176). K-MMSE noted significant improvements in place orientation (*p* = 0.0155), while a reduction in the values of K-MMSE temporal orientation (*p* = 0.3306) and visual CPT false alarm error (*p* = 0.0772) noted without any significant difference. Collectively, these findings may indicate a favorable trend toward improved SOD and cognitive outcomes following 40 mg PCO supplementation.

#### 3.2.4. Changes in HDL-C, Antioxidant SOD, and Selected Cognitive Outcomes from Baseline to Week 12

Table 5 shows changes in HDL-C, antioxidant SOD, and cognitive outcomes from baseline to week 12 across the placebo, 20 mg PCO, and 40 mg PCO groups. A post hoc exploratory sensitivity analysis included 56 participants (placebo, *n* = 19; 20 mg PCO, *n* = 18; 40 mg PCO, *n* = 19). Changes from baseline to week 12 showed a favorable pattern for several outcomes following 40 mg PCO supplementation. TNF-α and IL-6 decreased from baseline to Week 12 in both PCO groups, with greater numerical reductions observed in the 40 mg PCO group (−15.9 [*p* = 0.4756] and −2.1 pg/mL [*p* = 0.1639], respectively). In contrast, both inflammatory markers showed an arithmetic increase from baseline to Week 12 in the placebo group, indicating an unfavorable inflammatory trend. But these changes were not statistically significant within or between groups. The considerable inter-individual variability, particularly in TNF-α responses at 40 mg PCO, may have contributed to the lack of statistical significance (Supplementary Table S1). HDL-C significantly decreased in the placebo group (−3.7 ± 6.7 mg/dL, *p* = 0.0245), whereas slight increments were observed in the PCO 20 mg (+0.78 ± 5.1 mg/dL) and 40 mg (+2.2 ± 6.7 mg/dL) groups. Notably, significant between-group differences were observed (*p* = 0.0124 and *p* = 0.0142), indicating a favorable change in HDL-C in the 20 mg and 40 mg PCO groups compared with the placebo. Antioxidant SOD activity significantly increased in the 40 mg PCO group, with a mean change of +0.16 ± 0.25 U/mL, *p* = 0.0109, whereas the changes in the 20 mg PCO group (+0.09 ± 0.31 U/mL, *p* = 0.2170 and placebo group (+0.08 ± 0.24 U/mL, *p*=0.1873) were not found to be statistically significant. The study observed significant within-group improvements in selected cognitive outcomes in the PCO groups; however, no significant differences were found in between-group comparisons or ANCOVA analyses. ADAS-Cog 11 language ability scores decreased significantly in both the 20 mg PCO group (−1.17 ± 1.92 points, *p* = 0.0194) and the 40 mg PCO group (−0.95 ± 1.58 points, *p* = 0.0176), whereas the placebo group showed a nonsignificant decrease (−0.79 ± 1.72 points, *p* = 0.0605). The reduction in ADAS-Cog 11 language scores among participants receiving PCO may indicate an improvement in language performance over the 12-week treatment period, as lower scores on this measure reflect better performance; however, no statistically significant between-group differences were observed Similarly, K-MMSE place orientation scores increased significantly in both PCO groups: +0.94 ± 0.87 points (*p* = 0.0003) in 20 mg PCO and +0.47 ± 0.77 points (*p* = 0.0155) in 40 mg PCO. The placebo group showed a slight, nonsignificant increase of +0.32 ± 0.89 points (*p* = 0.1374). PCO intake was associated with significant within-group improvements in place-orientation scores at both 20 mg and 40 mg groups, whereas the change in the placebo group was not significant. For Visual CPT false alarm errors, reductions were observed in all groups, reaching statistical significance in the 20 mg PCO group. The changes in the 40 mg PCO and placebo groups were not statistically significant. Therefore, the findings represent exploratory within-group changes and require further investigation into larger populations receiving longer-term PCO supplementation.

**Figure 2:**
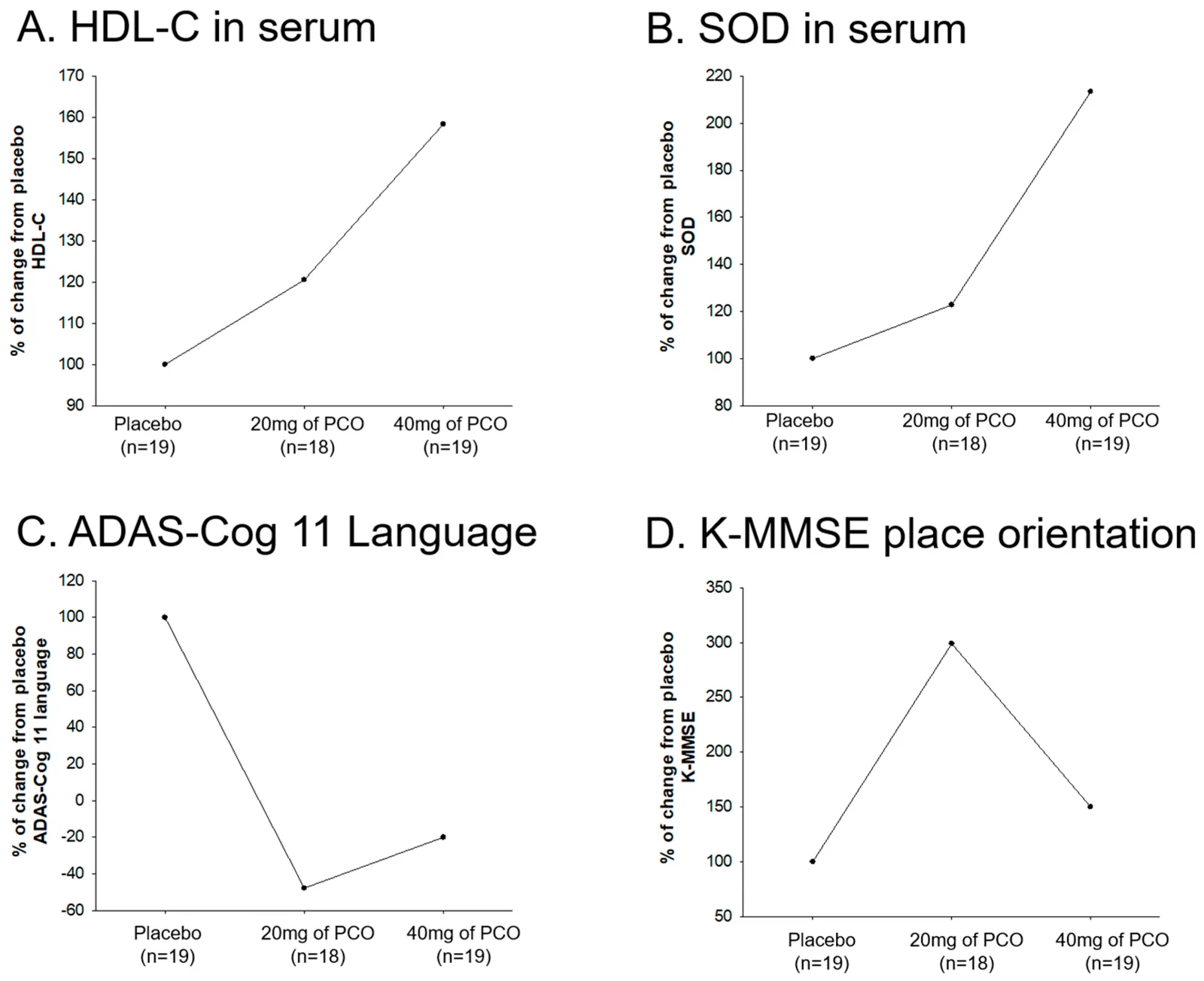
Visualized relative comparison of HDL-C, antioxidant SOD, and cognitive outcomes following 12 weeks of PCO supplementation.

As shown in Figure 2, the relative visualized comparison of HDL-C, SOD activity, ADAS-Cog 11 language scores, and K-MMSE scores in the PCO 20 mg and 40 mg groups after 12 weeks with the placebo group normalized to 100% as the reference. For HDL-C, the relative values indicate the percentage increase compared with the placebo reference. Similarly, SOD activity shows the relative elevation in the 20 and 40 mg (more than 2-fold) PCO groups, while the ADAS-Cog 11 language score demonstrates improvement in both PCO groups relative to placebo. The K-MMSE place-orientation score shows an elevation in the 20 mg PCO group, followed by a lower relative value in the 40 mg group, although it remained above the placebo reference. Thus, the figure provides an integrated visual representation of the direction and relative magnitude of changes across different parameters. These normalized values are presented primarily to facilitate visual comparison among the different PCO groups and can be interpreted descriptively.

## 4. Discussion

The present study investigated the effects of 12-week PCO supplementation on metabolic parameters (blood pressure, TC, TG, and other lipid profile), inflammatory biomarkers, antioxidant status SOD, and cognitive performance in individuals with SMD. The present study observed a slight increase in HDL-C levels (20 mg and 40 mg PCO) and numerical reductions in TC, TG, and LDL-C [119.4 to 115.4 mg/dL (−3.4%)], along with a significant increase in SOD activity in the 40 mg group (*p* = 0.0109), suggesting a favorable trend in lipid parameters and enhanced endogenous antioxidant defense. Although lipid profile changes from baseline to 12 weeks of consumption in 20 mg and 40 mg PCO groups were not statistically significant, the favorable increase in HDL-C and decrease in LDL-C with reduction in inflammatory markers may suggest a potential relationship with cognitive outcomes.

Impaired HDL cholesterol efflux and reduced anti-inflammatory activity in AD suggest that HDL dysfunction may be associated with disease severity [39]. HDL particles possess antioxidant and anti-inflammatory properties and are involved in protection against oxidative stress and inflammatory processes have been described in AD [40]. HDL functionality depends on its protein composition, which supports antioxidant, anti-inflammatory, and vasculo-protective activities. In AD, loss of protective HDL-associated proteins such as PON1/PON3 and GPx-3, together with increased prooxidant and proinflammatory mediators, may render HDL dysfunctional and contribute to vascular and brain pathology [41,42].

Lewis et al. in a transgenic mouse model of AD showed a protective role of apolipoprotein A-I (apoA-I)/HDL in cognitive function. Overexpression of human apoA-I increased plasma HDL-C and preserved learning and memory, while reducing cerebral amyloid angiopathy, glial activation, and Aβ-induced inflammatory responses. The finding suggested that an elevated apoA-I/HDL could help in preserving cognitive function, partially through a reduction in neuroinflammation and cerebrovascular amyloid pathology [43]. Additionally, clinical studies have reported lower circulating HDL-C and apoA-I levels in patients with AD, with lower apoA-I also associated with greater disease severity [44,45]. However, the present study assessed HDL-C concentration rather than HDL functionality. Previous studies of PCO have suggested that improved HDL functionality may enhance its antioxidant capacity [46,47], which could potentially contribute to favorable cognitive outcomes.

In the present study, IL-6 decreased from 6.4 to 4.3 pg/mL, and TNF-α decreased from 122.1 to 106.2 pg/mL in the 40 mg group, representing numerical reductions of approximately 33% and 13%, respectively; however, neither change was statistically significant (Table 4). Coincidentally, increased HDL-C and decreased LDL-C levels might contribute to the improvement of inflammatory markers in blood via reduction in oxLDL [48,49,50]. Nevertheless, chronic oxidative stress and inflammatory signaling have been implicated in neuronal dysfunction and cognitive decline, providing a biologically plausible connection between systemic metabolic/inflammatory status and cognitive performance.

Studies have shown that dyslipidemia and inflammation play a role in the development of neurodegeneration [51,52]. Lipids and lipoproteins may play a role in the neuropathology of AD. Changes in lipid parameters can influence the activity of enzymes related to the deposition of proteins such as Aβ and tau, which may accelerate the immune response in the periphery and CNS [53,54]. Moreover, cholesterol-associated genes are commonly linked with AD [55].

After 12 weeks of PCO supplementation, HDL levels increased numerically in both PCO groups (20 mg/day and 40 mg/day) compared with the placebo group (Table 5), with statistically significant differences observed in the between-group comparisons and baseline-adjusted ANCOVA. However, the within-group changes were not statistically significant. Likewise, antioxidant SOD status and inflammation, together with improvements in selected cognitive measures in the PCO group, suggest a plausible role of association between HDL/lipoprotein, oxidative stress, inflammation, and cognition. The study findings demand further investigation of a potential pathway. Particularly, HDL functionality was not assessed in the present study, and further research is needed to clarify these relationships among HDL functionality, oxidative stress, inflammation, and cognitive performance. Evidence from our research group and other colleagues has demonstrated antioxidant properties of PCO, including reduced LDL oxidative susceptibility in humans and inhibition of LDL oxidation in experimental HDL systems [23,56,57] without directly assessing neurological outcomes.

Our study results showed an enhancement of SOD activity within the 40 mg PCO group over 12 weeks (*p* = 0.0109), suggesting a potential increment of endogenous antioxidant defense. However, the corresponding between-group comparison was not statistically significant. SOD is a major component of the cellular antioxidant defense system and plays a central role in modulating superoxide-mediated oxidative stress. Oxidative stress and impaired antioxidant defenses have been implicated in cognitive aging and cognitive decline [58,59,60]. The present findings suggest that modulation of endogenous antioxidant defenses may contribute to its potential effects on age-related neurological dysfunction or age-related cognitive decline. Our findings provide preliminary clinical evidence of an antioxidant response to PCO in individuals with SMD and warrant further investigation in larger studies to determine whether modulation of antioxidant defenses contributes to the potential effects of PCO on age-related cognitive dysfunction.

We used ADAS-Cog 11, visual CPT, and K-MMSE scores to monitor cognitive performance in a Korean middle-aged population after intake of PCO at different doses. We have reported cognitive measures, changes in ADAS-Cog 11 language scores, K-MMSE place orientation, and visual CPT false alarm errors in Table 5. Cognitive measures showed significant within-group changes, including improvements in ADAS-Cog 11 language scores in the 20 mg PCO and 40 mg PCO groups and K-MMSE place orientation scores in both PCO groups, whereas no significant changes were observed in the placebo group. Visual CPT false alarm errors also significantly decreased in the 20 mg PCO group, while non-significant in the 40 mg PCO group. This difference might be due to the relatively small sample size and substantial interindividual variability of cognitive performance in the participants of the 40 mg PCO group. Additionally, the slightly broader age range among the participants may also have contributed to this variability. The present study provides preliminary evidence that PCO may have potential relevance to cognitive health in older adults with SMD. PCO was well tolerated, supporting further investigation of its potential role in maintaining cognitive function during aging. The study warrants further investigation of the potential relationship between antioxidant defense and cognitive performance in participants with SMD. Orientation to place is an important component of global cognitive assessment, is commonly included in cognitive assessment tools, and could be affected in vascular cognitive impairment. Previous studies have revealed poorer place-orientation performance in people with vascular dementia and have detected orientation deficits even before clinical dementia diagnosis [61,62]. In our study, K-MMSE place-orientation scores improved significantly in both the 20 mg and 40 mg PCO groups but not in the placebo group. Though our participants were not diagnosed with vascular dementia, this finding may highlight a potential benefit of PCO on orientation-related cognitive performance, which may be relevant to cognitive changes associated with vascular dysfunction [63] and warrants further clinical investigation in a sufficient number of participants.

It has been previously known that oxidative stress plays a detrimental role in cognitive decline and AD [58,64]. The brain is more susceptible than other organs as far as oxidative stress and lipid peroxidation are concerned. Neurons are composed of lipids, proteins, and nucleic acids, which are essential components of their cellular membranes, structural elements, and genetic machinery. These are excessively oxidized in conditions with AD owing to mitochondrial dysfunction, inflammation, and deposition of β-amyloid (Aβ) peptide [65]. Oxidative stress can also be involved in AD or dementia by assisting beta-amyloid accumulation, Tau hyperphosphorylation, and later damaging the synapses and entire neuron [66].

Under conditions of oxidative stress, excessive production of ROS overcomes the antioxidant defense system, resulting in an imbalance between ROS generation and antioxidant capacity. Oxidative stress and impaired antioxidant defenses have been associated with cognitive impairment and mild cognitive impairment, supporting the biological relevance of examining antioxidant status in populations with subjective cognitive complaints [67,68,69]. This redox imbalance can activate stress-related signaling pathways and contribute to neuronal dysfunction and cognitive decline [70]. Alterations in SOD activity have also been associated with neurodegenerative disorders, including AD [71,72].

Sánchez et al. provided supportive evidence in humans for the potential neurological relevance of PCO, while our findings focused on cognitive outcomes in a less clinically impaired population. The former study reported observations in patients with ischemic stroke and previous transient ischemic attacks in which consumption of PCO (20 mg/day) combined with aspirin was associated with progressive enhancement in neurological outcomes and lowered recurrent cerebrovascular events; our findings suggest that PCO may have potential effects on selected cognitive outcomes, although the present study was conducted in individuals with subjective memory complaints rather than patients with established cerebrovascular disease [73].

This study has certain limitations. First, the refined cognitive analysis included a relatively small sample of 56 participants and was based on post hoc selection criteria, including the exclusion of statin users and restriction to a K-MMSE score range of 18–26. Although these criteria were intended to reduce heterogeneity in baseline cognitive performance and address potential ceiling effects, they do not constitute a validated diagnostic definition of SMD. Additionally, the sensitivity analyses in the original population were not performed, and therefore findings should be interpreted as exploratory and may have limited robustness and generalizability across cognitive domains. Second, although we assessed multiple ADAS-Cog 11, K-MMSE, and Visual CPT parameters, the refined analysis presented only selected cognitive domains, which may introduce outcome-selection and multiple-comparison considerations. Third, enhancement of HDL quality/functionality and molecular pathways underlying the observed SOD response, including neuroinflammatory and other signaling pathways, were not directly assessed. Future studies are required to elucidate the relationship between HDL quality, antioxidant capacity, and cognitive function, as well as to clarify the underlying mechanisms linking HDL functionality to cognitive health. Finally, several favorable cognitive and inflammatory changes were observed within groups but did not differ significantly between groups. Overall, the findings suggest that PCO may have biological effects relevant to antioxidant defense and selected aspects of cognitive performance, but confirmation in a larger, adequate number of participants incorporating direct molecular and neuroinflammatory measures is required.

## 5. Conclusions

In this 12-week randomized, double-blinded, placebo-controlled, multicenter RAYDELP1 study, PCO supplementation was associated with improvements in cognitive outcomes and antioxidant status in adults with SMD. The 40 mg PCO group showed a significant increase in SOD activity, along with significant improvements in ADAS-Cog 11 language scores and K-MMSE place orientation. Also, a slight increase in HDL-C and a reduction in LDL-C and inflammatory markers (IL-6 and TNF-α) in the 40 mg group were observed; however, these changes were not statistically significant. Therefore, the findings suggest that PCO may have beneficial effects on antioxidant defense and selected aspects of cognitive performance, with a possible contribution from favorable lipoprotein and inflammatory changes. Although differences between groups in lipid and inflammatory markers were not significant, the observed within-group changes suggest that longer-term PCO supplementation may warrant investigation for potential effects on these pathways and their relationship with cognitive decline.

## Figures and Tables

**Table 1:** Baseline demographic characteristics of participants in the RAYDELP1 trial.

Baseline Characteristics	Placebo (*n* = 19)	20 mg PCO (*n* = 18)	40 mg PCO (*n* = 19)	*p*-Value
Age (years)	61.84 ± 5.32	63.11 ± 6.94	63.53 ± 7.76	0.8769
Age range	55–73	55–77	55–81	
Sex, *n* (%)				0.7735
Male, *n*, age range, (%)	5, 59–73, (26.32)	3, 63–77, (16.67)	4, 66–81, (21.05)	
Female, *n*, age range, (%)	14, 55–71, (73.68)	15, 55–73, (83.33)	15, 55–77, (78.95)	
Height (cm)	159.00 ± 7.80	159.12 ± 6.13	159.84 ± 6.51	0.8390
Weight (kg)	63.18 ± 9.41	61.52 ± 8.60	60.55 ± 7.55	0.6350
BMI (kg/m^2^)	24.86 ± 3.18	24.02 ± 2.76	23.64 ± 2.70	0.4185
Education level, *n* (%)				0.7805
No formal education or elementary school dropout	0 (0.00)	0 (0.00)	0 (0.00)	
Elementary school graduate or middle school dropout	3 (15.79)	1 (5.56)	0 (0.00)	
Middle school graduate or high school dropout	1 (5.26)	1 (5.56)	1 (5.26)	
High school graduate or university dropout	8 (42.11)	9 (50.00)	9 (47.37)	
College/associate degree or university graduate	6 (31.58)	7 (38.89)	8 (42.11)	
Graduate school enrollment or graduation	1 (5.26)	0 (0.00)	1 (5.26)	

Values are presented as mean ± SD or *n* (%). *p*-values were calculated for the 56-participant analysis population. Continuous variables were compared using the Kruskal–Wallis test for age and height and one-way ANOVA for weight; categorical variables were compared using the chi-square test. Abbreviations: BMI, body mass index; PCO, policosanol.

**Table 2:** Metabolic, inflammatory biomarkers, and cognitive characteristics of participants receiving placebo (*n* = 19).

Metabolic, Inflammatory Biomarkers, and Cognitive Characteristics	Baseline	Week 12	*p*-Value
Systolic blood pressure (mmHg)	133.4 ± 17.6	123.6 ± 12.6	0.0054
Diastolic blood pressure (mmHg)	84.6 ± 9.7	76.4 ± 9.4	0.0008
Total cholesterol (mg/dL)	192.5 ± 35.6	186.2 ± 26.9	0.2809
Triglyceride (mg/dL)	147.5 ± 103.2	149.1 ± 101.4	0.9465
High-density lipoprotein (HDL) cholesterol (mg/dL)	54.3 ± 14.5	50.5 ± 15.6	0.0245
Low-density lipoprotein (LDL) cholesterol (mg/dL)	110.5 ± 30.4	109.3 ± 27.3	0.7801
Triglyceride/High-density lipoprotein cholesterol	3.2 ± 3.3	3.5 ± 3.1	0.7120
LDL-cholesterol/HDL-cholesterol	2.1 ± 0.7	2.3 ± 0.7	0.0146
High-density lipoprotein cholesterol/Total cholesterol (%)	28.8 ± 7.7	27.2 ± 7.8	0.1302
Glucose	103.2 ± 12.9	99.0 ± 11.5	0.0107
Glucose/High-density lipoprotein cholesterol	2.0 ± 0.5	2.1 ± 0.6	0.2642
Creatinine	0.7 ± 0.1	0.7 ± 0.1	0.3382
Uric acid	4.5 ± 1.0	4.6 ± 1.1	0.2359
Alkaline phosphatase	71.2 ± 23.7	69.4 ± 22.9	0.3393
Alanine aminotransferase	26.8 ± 14.0	22.8 ± 11.5	0.1702
Aspartate Aminotransferase	26.5 ± 8.5	25.5 ± 6.4	0.4735
Albumin	4.2 ± 0.2	4.1 ± 0.3	0.0880
Blood urea nitrogen	15.4 ± 2.8	16.6 ± 4.8	0.2820
Bilirubin	0.7 ± 0.3	0.6 ± 0.4	0.3352
Interleukin-6	1.2 ± 1.2	1.3 ± 1.2	0.7346
Tumor necrosis factor (TNF)-α	164.3 ± 676.4	169.4 ± 647.2	0.5860
Superoxide dismutase	1.2 ± 0.6	1.2 ± 0.6	0.1873
ADAS-Cog11 language	1.74 ± 1.37	0.95 ± 0.85	0.0605
Visual CPT false alarm errors	8.37 ± 13.04	2.79 ± 2.30	0.0593
K-MMSE temporal orientation	4.47 ± 0.61	4.74 ± 0.45	0.1353
K-MMSE place orientation	4.37 ± 0.76	4.68 ± 0.58	0.1374

Values are presented as mean ± SD. *p*-values represent within-group comparisons between baseline and Week 12 using paired *t*-tests. Abbreviations: ADAS-Cog, Alzheimer’s disease assessment scale; Visual CPT, visual continuous performance test; K-MMSE, Korean-mini mental state examination.

**Table 3:** Metabolic, inflammatory biomarkers, and cognitive characteristics of participants receiving PCO 20 mg (*n* = 18).

Metabolic, Inflammatory Biomarkers, and Cognitive Characteristics	Baseline	Week 12	*p*-Value
Systolic blood pressure (mmHg)	129.8 ± 15.5	125.1 ± 14.4	0.2021
Diastolic blood pressure (mmHg)	77.2 ± 6.7	76.6 ± 7.9	0.7216
Total cholesterol (mg/dL)	198.6 ± 44.2	201.0 ± 46.9	0.4820
Triglyceride (mg/dL)	114.3 ± 66.2	116.5 ± 49.0	0.8651
High-density lipoprotein (HDL) cholesterol (mg/dL)	56.8 ± 13.2	57.6 ± 13.5	0.5267
Low-density lipoprotein (LDL) cholesterol (mg/dL)	116.2 ± 42.5	120.2 ± 42.9	0.3966
Triglyceride/High-density lipoprotein cholesterol	2.2 ± 1.7	2.1 ± 1.1	0.8467
LDL-cholesterol/HDL-cholesterol	2.2 ± 1.0	2.2 ± 1.1	0.6519
High-density lipoprotein cholesterol/Total cholesterol (%)	30.1 ± 9.8	30.3 ± 10.1	0.7843
Glucose	95.7 ± 7.6	101.8 ± 20.9	0.2039
Glucose/High-density lipoprotein cholesterol	1.7 ± 0.4	1.8 ± 0.5	0.3511
Creatinine	0.7 ± 0.1	0.7 ± 0.1	0.4252
Uric acid	4.5 ± 1.5	4.6 ± 1.3	0.2914
Alkaline Phosphatase	55.8 ± 14.3	57.2 ± 14.3	0.3477
Alanine Aminotransferase	24.2 ± 10.9	23.9 ± 14.7	0.8809
Aspartate Aminotransferase	28.1 ± 7.0	28.1 ± 8.9	1.0000
Albumin	4.3 ± 0.2	4.3 ± 0.1	1.0000
Blood urea nitrogen	15.3 ± 3.4	15.7 ± 3.1	0.6622
Bilirubin	0.7 ± 0.1	0.7 ± 0.2	0.6207
Interleukin-6	2.0 ± 3.4	1.2 ± 2.0	0.3183
Tumor necrosis factor (TNF)-α	15.3 ± 36.3	7.8 ± 13.6	0.2118
Superoxide dismutase	1.0 ± 0.7	1.1 ± 0.8	0.2170
ADAS-Cog11 language	1.67 ± 2.25	0.50 ± 0.86	0.0194
Visual CPT false alarm errors	6.11 ± 8.47	2.28 ± 2.32	0.0357
K-MMSE temporal orientation	4.17 ± 0.99	4.50 ± 0.92	0.1872
K-MMSE place orientation	3.94 ± 0.80	4.89 ± 0.32	0.0003

Values are presented as mean ± SD. *p*-values represent within-group comparisons between baseline and week 12 using paired *t*-tests. Abbreviations: ADAS-Cog, Alzheimer’s disease assessment scale; Visual CPT, visual continuous performance test; K-MMSE, Korean mini-mental state examination.

**Table 4:** Metabolic, inflammatory biomarkers, and cognitive characteristics of participants receiving PCO 40 mg (*n* = 19).

Metabolic, Inflammatory Biomarkers, and Cognitive Characteristics	Baseline	Week 12	*p*-Value
Systolic blood pressure (mmHg)	123.8 ± 14.6	119.6 ± 15.9	0.1674
Diastolic blood pressure (mmHg)	75.0 ± 9.4	75.0 ± 9.7	1.0000
Total cholesterol (mg/dL)	203.9 ± 27.9	200.0 ± 38.5	0.5294
Triglyceride (mg/dL)	128.0 ± 100.8	115.6 ± 58.5	0.4289
High-density lipoprotein (HDL) cholesterol (mg/dL)	59.8 ± 14.5	62.0 ± 16.7	0.1673
Low-density lipoprotein (LDL) cholesterol (mg/dL)	119.4 ± 33.8	115.4 ± 36.8	0.4721
Triglyceride/High-density lipoprotein cholesterol	2.7 ± 3.2	2.2 ± 1.7	0.3138
LDL-cholesterol/HDL-cholesterol	2.1 ± 0.8	1.9 ± 0.7	0.3016
High-density lipoprotein cholesterol/Total cholesterol (%)	29.6 ± 7.0	31.5 ± 8.4	0.0701
Glucose	93.6 ± 12.6	96.8 ± 12.7	0.1870
Glucose/High-density lipoprotein cholesterol	1.6 ± 0.5	1.7 ± 0.6	0.6543
Creatinine	0.7 ± 0.2	0.7 ± 0.2	0.9107
Uric acid	4.7 ± 1.3	4.8 ± 1.2	0.7108
Alkaline phosphatase	60.0 ± 14.7	61.6 ± 18.4	0.3178
Alanine aminotransferase	22.0 ± 20.5	19.1 ± 5.8	0.5081
Aspartate aminotransferase	25.9 ± 12.1	23.7 ± 4.3	0.3914
Albumin	4.2 ± 0.2	4.2 ± 0.2	0.5778
Blood urea nitrogen (BUN)	14.4 ± 4.2	16.9 ± 5.0	0.0380
Bilirubin	0.7 ± 0.2	0.7 ± 0.2	0.7429
Interleukin (IL)-6	6.4 ± 20.0	4.3 ± 13.8	0.1639
Tumor necrosis factor (TNF)-α	122.1 ±513.1	106.2 ± 420.6	0.4756
Superoxide dismutase (SOD)	0.8 ± 0.4	0.9 ± 0.4	0.0109
ADAS-Cog11 language	1.84 ± 1.54	0.89 ± 1.15	0.0176
Visual CPT false alarm errors	9.63 ± 16.53	3.42 ± 3.72	0.0772
K-MMSE temporal orientation	4.11 ± 0.88	4.37 ± 0.90	0.3306
K-MMSE Place orientation	4.26 ± 0.73	4.74 ± 0.45	0.0155

Values are presented as mean ± SD. *p*-values represent within-group comparisons between baseline and week 12 using paired *t*-tests. Abbreviations: ADAS-Cog, Alzheimer’s disease assessment scale; Visual CPT, visual continuous performance test; K-MMSE, Korean-mini mental state examination.

**Table 5:** Changes in HDL-C, antioxidant SOD, and selected cognitive outcomes from baseline to week 12.

Outcome	Placebo (*n* = 19)	20 mg PCO (*n* = 18)	40 mg PCO (*n* = 19)	Between-Group *p*	ANCOVA *p*
HDL-C (mg/dL)	−3.79 ± 6.73 *p* = 0.0245	+0.78 ± 5.11 *p* = 0.5267	+2.21 ± 6.70 *p* = 0.1673	0.0124	0.0142
SOD activity (U/mL)	+0.08 ± 0.24 (*p* = 0.1873)	+0.09 ± 0.31 (*p* = 0.2170)	+0.16 ± 0.25 (*p* = 0.0109) *	0.5802	0.5753
ADAS-Cog 11 language ability score (points)	−0.79 ± 1.72 (*p* = 0.0605)	−1.17 ± 1.92 (*p* = 0.0194) *	−0.95 ± 1.58 (*p* = 0.0176) *	0.8547	0.3221
K-MMSE place orientation (points)	+0.32 ± 0.89 (*p* = 0.1374)	+0.94 ± 0.87 (*p* = 0.0003) *	+0.47 ± 0.77 (*p* = 0.0155) *	0.0958	0.3067
Visual CPT/false-alarm errors	−5.58 ± 12.08 (*p* = 0.0593)	−3.83 ± 7.13 (*p* = 0.0357) *	−6.21 ± 14.44 (*p* = 0.0772)	0.7604	0.6594

* *p* < 0.05 versus baseline within the same treatment group (paired *t*-test). Values are presented as mean ± SD change from baseline to week 12. Between-group *p*-values represent comparisons among the placebo, 20 mg, and 40 mg PCO groups; ANCOVA *p*-values represent baseline-adjusted comparisons. Abbreviations: HDL-C, high-density lipoprotein cholesterol; SOD, superoxide dismutase; ADAS-Cog 11, Alzheimer’s disease assessment scale; Visual CPT, visual continuous performance test; K-MMSE, Korean mini-mental state examination.

## Data Availability

The datasets generated and analysed during the current study are available by request.

## Supplementary Materials

The following supporting information can be downloaded at: https://www.mdpi.com/article/10.3390/nu18213496/s1, Supplementary Table S1: Changes in inflammatory markers from baseline to Week 12. Supplementary Table S2: Exclusion criteria for study participation. Supplementary Table S3: Information related to the batch number of the used policosanol (PCO). Supplementary Table S4: Details of the plant registration form.

## Clinical Trial Registration

The study was retrospectively registered with the Clinical Research Information Service (CRIS, Osong, Republic of Korea), under registration number KCT0012761. The registration application was submitted on 2 October 2026, and registration was completed on 11 October 2026. https://cris.nih.go.kr/cris/search/detailSearch.do?search_lang=E&focus=reset_12&search_page=L&pageSize=10&page=undefined&seq=35230&status=5&seq_group=35058 (accessed on 19 October 2026).

## Abbreviations

The following abbreviations are used in this manuscript:

PCO	Policosanol
SMD	Subjective memory decline
SOD	Superoxide dismutase
ROS	Reactive oxygen species
HDL-C	High-density lipoprotein cholesterol
LDL-C	Low-density lipoprotein cholesterol
ADAS-Cog 11	Alzheimer’s Disease Assessment Scale–Cognitive Subscale, 11-item
K-MMSE	Korean Mini-Mental State Examination
CPT	Continuous Performance Test
SBP	Systolic blood pressure
DBP	Diastolic blood pressure
TC	Total cholesterol
TG	Triglycerides
BUN	Blood urea nitrogen
ALP	Alkaline phosphatase
ALT	Alanine aminotransferase
AST	Aspartate aminotransferase
IL-6	Interleukin-6
TNF-α	Tumor necrosis factor-alpha
