# Supplementary material for: Efficacy and Safety of Cuban Policosanol on Cognitive Function and Antioxidant Status in Adults with Subjective Memory Decline: A 12-Week Randomized, Double-Blinded, Placebo-Controlled Multicenter Trial
Source: Nutrients. 2026 Oct 24;18(21):3496. doi: 10.3390/nu18213496 (PMC13764964; doi:10.3390/nu18213496)
Supplement: Supplementary file 1 [file nutrients-18-03496-s001.zip › nutrients-4615172-supplementary.pdf]

Supplementary Material

Supplementary Table S1. Changes in inflammatory markers from baseline to Week 12

Inflammatory markers	Placebo (n=19)	20 mg PCO (n=18)	40 mg PCO (n=19)	Between-group *p*	ANCOVA *p*
**TNF-α (pg/mL)**	+5.12 ± 40.21 (*p*=0.5860)	−7.49 ± 24.48 (*p*=0.2118)	−15.92 ± 95.22 (*p*=0.4756)	0.5762	0.0877
**IL-6 (pg/mL)**	+0.09 ± 1.17 (*p*=0.7346)	−0.80 ± 3.28 (*p*=0.3183)	−2.15 ± 6.44 (*p*=0.1639)	0.2711	0.5243

*** p<0.05 versus baseline within the same treatment group (paired t-test).** Values are presented as mean ± SD change from baseline to Week 12. Between-group p-values represent comparisons among the three treatment groups; ANCOVA p-values represent baseline-adjusted comparisons.

Supplementary Table S2. Exclusion criteria for study participation.

S. No	Exclusion criteria
1	Participants who are currently receiving treatment for severe diseases of the immune system, respiratory system, gastrointestinal/hepatobiliary system, renal/urinary system, nervous system, musculoskeletal system, infectious diseases, malignancies, or other serious medical conditions. However, participation may be permitted at the investigator's discretion based on the participant's clinical condition.
2	Participants with a current psychiatric disorder, such as depression (based on DSM-5[Diagnostic and Statistical Manual of Mental Disorders-V] criteria), schizophrenia, alcohol dependence, or drug dependence.
3	Participants with a disease associated with cognitive impairment, such as dementia (based on DSM-5[Diagnostic and Statistical Manual of Mental Disorders-V] criteria) or Parkinson's disease.
4	Participants with a history of head trauma accompanied by loss of consciousness or cardiovascular/cerebrovascular disease, including angina pectoris, myocardial infarction, transient ischemic attack, or acute stroke, within 6 months prior to Visit 1.
5	Participants who have used medications that may affect cognitive function, including antipsychotics, medications for degenerative diseases, cerebral function-enhancing agents, or tricyclic antidepressants, within 4 weeks prior to Visit 1.
6	Participants receiving vitamin E at a daily dose of 400 IU or more, or for whom a reduction in the vitamin E dose is considered not feasible.
7	Participants who have received estrogen replacement therapy, excluding topical preparations, within 2 months prior to Visit 1.

8	Participants who have taken health functional foods intended to improve cognitive function within 2 weeks prior to Visit 1.
9	Participants with thyroid disease and a TSH level of ≤0.1 μIU/mL or ≥10 μIU/mL.
10	Participants with a creatinine level ≥2 times the upper limit of normal (ULN) at the study site.
11	Participants with AST (GOT) or ALT (GPT) levels ≥3 times the upper limit of normal (ULN) at the study site.
12	Participants with uncontrolled hypertension, defined as systolic blood pressure ≥160 mmHg or diastolic blood pressure ≥100 mmHg, measured after at least 10 minutes of rest.
13	Participants with uncontrolled diabetes mellitus, defined as a fasting blood glucose level ≥180 mg/dL.
14	Participants with known hypersensitivity or allergy to any ingredient of the investigational product used in this clinical trial.
15	Participants who are pregnant or breastfeeding, or who plan to become pregnant during the clinical trial period.
16	Participants who have participated in another interventional clinical trial, including a human application trial, within 3 months prior to Visit 1, or who plan to participate in another interventional clinical trial after initiation of this clinical trial.
17	Participants who are considered inappropriate for participation in the clinical trial by the investigator for any other reason.

Supplementary Table S3. Information related to the batch number of the used policosanol (PCO).

[시생산 기록서]

	R&I			
	담당	파트장	팀장	임원
결재				

번호 : 2024-146 담당자 : 이은정

고객사	임상용 폴리코사놀20mg	MKT담당	이윤제
제품명	폴리코사놀 20mg	제품유형	건강기능식품
제형	제피정제	펀치	8파이 원형
성상		섭취방법	1일1회, 1회2정/병포장
목적	임상용	시생산일자	2024-12-17

* 성분배합비율

단위중량		200 mg	총량	3.0 kg		
**No.**	**원료 Code**	**원 료 명**	**배합비율**	**단위중량**	**투입량(g)**	**비 고**
1	100010684	폴리코사놀(사탕수수왁스)/우리레인보우/사급	5.5560 %	11.1120 mg	166.6800	
2	100012308	유당	54.0303 %	108.0606 mg	1,620.9090	
3	100001586	결정셀룰로오스	35.6137 %	71.2274 mg	1,068.4110	
4	100000684	CMC칼슘/카르복시메틸셀룰로오스칼슘	1.0000 %	2.0000 mg	30.0000	
5	100001395	스테아린산마그네슘	0.8000 %	1.6000 mg	24.0000	
		[코팅]				
6	100000645	HPMC(히드록시프로필메틸셀룰로스)	2.2620 %	4.5240 mg	67.8600	
7	100001270	글리세린지방산에스테르	0.0750 %	0.1500 mg	2.2500	
8	100000761	이산화티타늄	0.1430 %	0.2860 mg	4.2900	
9	100000122	치자청색소	0.5200 %	1.0400 mg	15.6000	
11	100000563	발효주정	3.5500 %	7.1000 mg	106.5000	
		합 계	100.0000 %	200.0000 mg	3000.0000	

* 제조방법

No.	공 정 내 용	확인자
1	**1차 혼합**<br>이산화규소, 스테아린산마그네슘을 제외한 모든 원료를 20mesh에 체과하여 혼합한다.	
2	**2차 혼합**<br>위의 혼합물에 이산화규소, 스테아린산마그네슘을 20mesh에 체과하여 혼합한다.	
3	**타정**	
4	**코팅**	

*주의 사항 및 특이사항

Pilot Production Record

Record No.: 2024-146 Person in Charge: Lee Eun-jeong MKT Contact: Lee Yun-je

Field	Source entry / English rendering
Customer	Clinical-use Policosanol 20 mg
Product name	Policosanol 20 mg
Product type	Health functional food
Dosage form	Coated tablet
Punch	8 mm, round
Appearance	Blank in source
Directions for use	Once daily, 2 tablets per dose / bottle packaging
Purpose	For clinical trial use
Pilot production date	2024-12-17

Ingredient composition ratio

Unit weight: 200 mg Total batch: 3.0 kg

No.	Raw material code	Raw material name (English rendering)	Ratio	Unit weight	Input amount (g)	Remarks
1	100010684	Policosanol (sugarcane wax) / Woori Rainbow / supplied material	5.5560%	11.1120 mg	166.6800	
2	100012308	Lactose	54.0303%	108.0606 mg	1,620.9090	
3	100001586	Crystalline cellulose	35.6137%	71.2274 mg	1,068.4110	
4	100000684	CMC calcium / calcium carboxymethylcellulose	1.0000%	2.0000 mg	30.0000	
5	100001395	Magnesium stearate	0.8000%	1.6000 mg	24.0000	
6	100000645	HPMC (hydroxypropyl methylcellulose)	2.2620%	4.5240 mg	67.8600	Coating
7	100001270	Glycerol fatty acid ester	0.0750%	0.1500 mg	2.2500	Coating
8	100000761	Titanium dioxide	0.1430%	0.2860 mg	4.2900	Coating
9	100000122	Gardenia blue color	0.5200%	1.0400 mg	15.6000	Coating
11	100000563	Fermented ethanol	3.5500%	7.1000 mg	106.5000	Coating
		Total	100.0000%	200.0000 mg	3000.0000	

Manufacturing method

No.	Step	Process description
1	First mixing	All raw materials except silicon dioxide and magnesium stearate are passed through a 20-mesh sieve and mixed.
2	Second mixing	Silicon dioxide and magnesium stearate are passed through a 20-mesh sieve and mixed with the mixture above.
3	Tableting	No further details entered in source.
4	Coating	No further details entered in source.

Precautions / special notes: blank in source.

COSMAXBIO

* 제조내용 및 품질 체크사항

No.	공 정 내 용
1	혼합 [혼합실 : 19~25℃ / 60% 이하]<br>온 / 습도 : 20.5℃ / 27%<br>사별체 사이즈 : 20 mesh<br>[중량기록지]
2	타정 조건 [타정실 : 19~25℃ / 60% 이하]
3	결과<br>[최종 타정규격]

타정실 / 설비	3실/PKT-30
온 / 습도	23.1/31%
레일 사이즈 (셋팅/실제)	8.5mm
피더 속도 (10~50 RPM)	30
타정 속도 (20~60 RPM)	30

항 목	설정 기준	결 과
경도 (kgf)	3 이상	3-4
두께 (mm)	3.4~3.8	3.4-3.5
중량 (mg)	194±3%(189-199)	195-196
마손도 (%) (20T,25RPM,100회)	1.0% 이하	적합
붕해 (분)	50분 이하	적합

Manufacturing Details and Quality Check Items

No.	Item	Source entry / English rendering
1	Mixing	Mixing room: 19–25°C / relative humidity ≤60%<br>Temperature / humidity: 20.5°C / 27%<br>Sieve size: 20 mesh<br>[Weight record sheet] (blank area in source)
2	Tableting conditions	Tableting room: 19–25°C / relative humidity ≤60%<br>Tableting room / equipment: Room 3 / PKT-30<br>Temperature / humidity: 23.1 / 31%<br>Rail size (setting/actual): 8.5 mm<br>Feeder speed (10–50 RPM): 30<br>Tableting speed (20–60 RPM): 30

3. Results — Final tablet specifications

Item	Acceptance criterion	Result
Hardness (kgf)	≥3	3–4
Thickness (mm)	3.4–3.8	3.4–3.5
Weight (mg)	194 ± 3% (189–199)	195–196
Friability (%) (20 tablets, 25 RPM, 100 revolutions)	≤1.0%	Conforms
Disintegration (minutes)	≤50 minutes	Conforms

[시생산 기록서]

	R&I			
	담당	파트장	팀장	임원
결재				

번호 : 2024-146 담당자 : 이은정

고객사	임상용 폴리코사놀40mg	MKT담당	이윤제
제품명	폴리코사놀 40mg	제품유형	건강기능식품
제형	제피정제	펀치	8파이 원형
성상		섭취방법	1일1회, 1회2정/병포장
목적	임상용	시생산일자	2024-12-17

* 성분배합비율

단위중량		200 mg	총량	3.0 kg		
**No.**	**원료 Code**	**원 료 명**	**배합비율**	**단위중량**	**투입량(g)**	**비 고**
1	100010684	폴리코사놀(사탕수수왁스)/우리레인보우/사급	11.1120 %	22.2240 mg	333.3600	
2	100012308	유당	54.0303 %	108.0606 mg	1,620.9090	
3	100001586	결정셀룰로오스	30.0577 %	60.1154 mg	901.7310	
4	100000684	CMC칼슘/카르복시메틸셀룰로오스칼슘	1.0000 %	2.0000 mg	30.0000	
5	100001395	스테아린산마그네슘	0.8000 %	1.6000 mg	24.0000	
		[코팅]				
6	100000645	HPMC(히드록시프로필메틸셀룰로스)	2.2620 %	4.5240 mg	67.8600	
7	100001270	글리세린지방산에스테르	0.0750 %	0.1500 mg	2.2500	
8	100000761	이산화티타늄	0.1430 %	0.2860 mg	4.2900	
9	100000122	치자청색소	0.5200 %	1.0400 mg	15.6000	
11	100000563	발효주정	3.5500 %	7.1000 mg	106.5000	
		합 계	100.0000 %	200.0000 mg	3000.0000	

* 제조방법

No.	공 정 내 용	확인자
1	**1차 혼합**<br>이산화규소, 스테아린산마그네슘을 제외한 모든 원료를 20mesh에 체과하여 혼합한다.	
2	**2차 혼합**<br>위의 혼합물에 이산화규소, 스테아린산마그네슘을 20mesh에 체과하여 혼합한다.	
3	**타정**	
4	**코팅**	

*주의 사항 및 특이사항

Pilot Production Record

Record No.: 2024-146 Person in Charge: Lee Eun-jeong MKT Contact: Lee Yun-je

Field	Source entry / English rendering
Customer	Clinical-use Policosanol 40 mg
Product name	Policosanol 40 mg
Product type	Health functional food
Dosage form	Coated tablet
Punch	8 mm, round
Appearance	Blank in source
Directions for use	Once daily, 2 tablets per dose / bottle packaging
Purpose	For clinical trial use
Pilot production date	2024-12-17

Ingredient composition ratio

Unit weight: 200 mg Total batch: 3.0 kg

No.	Raw material code	Raw material name (English rendering)	Ratio	Unit weight	Input amount (g)	Remarks
1	100010684	Policosanol (sugarcane wax) / Woori Rainbow / supplied material	11.1120%	22.2240 mg	333.3600	
2	100012308	Lactose	54.0303%	108.0606 mg	1,620.9090	
3	100001586	Crystalline cellulose	30.0577%	60.1154 mg	901.7310	
4	100000684	CMC calcium / calcium carboxymethylcellulose	1.0000%	2.0000 mg	30.0000	
5	100001395	Magnesium stearate	0.8000%	1.6000 mg	24.0000	
6	100000645	HPMC (hydroxypropyl methylcellulose)	2.2620%	4.5240 mg	67.8600	Coating
7	100001270	Glycerol fatty acid ester	0.0750%	0.1500 mg	2.2500	Coating
8	100000761	Titanium dioxide	0.1430%	0.2860 mg	4.2900	Coating
9	100000122	Gardenia blue color	0.5200%	1.0400 mg	15.6000	Coating
11	100000563	Fermented ethanol	3.5500%	7.1000 mg	106.5000	Coating
		Total	100.0000%	200.0000 mg	3000.0000	

Manufacturing method

No.	Step	Process description
1	First mixing	All raw materials except silicon dioxide and magnesium stearate are passed through a 20-mesh sieve and mixed.
2	Second mixing	Silicon dioxide and magnesium stearate are passed through a 20-mesh sieve and mixed with the mixture above.
3	Tableting	No further details entered in source.
4	Coating	No further details entered in source.

Precautions / special notes: blank in source.

COSMAXBIO

* 제조내용 및 품질 체크사항

No.	공 정 내 용
1	혼합 [혼합실 : 19~25℃ / 60% 이하]<br>온 / 습도 : 20.5℃ / 27%<br>사별체 사이즈 : 20 mesh<br>[중량기록지]
2	타정 조건 [타정실 : 19~25℃ / 60% 이하]
3	결과<br>[최종 타정규격]

타정실 / 설비	3실/PKT-30
온 / 습도	23.1/31%
레일 사이즈 (셋팅/실제)	8.5mm
피더 속도 (10~50 RPM)	30
타정 속도 (20~60 RPM)	30

항 목	설정 기준	결 과
경도 (kgf)	3 이상	3-4
두께 (mm)	3.4~3.8	3.4-3.5
중량 (mg)	194±3%(189-199)	195-196
마손도 (%) (20T,25RPM,100회)	1.0% 이하	적합
붕해 (분)	50분 이하	적합

Manufacturing Details and Quality Check Items

No.	Item	Source entry / English rendering
1	Mixing	Mixing room: 19–25°C / relative humidity ≤60%<br>Temperature / humidity: 20.5°C / 27%<br>Sieve size: 20 mesh<br>[Weight record sheet] (blank area in source)
2	Tableting conditions	Tableting room: 19–25°C / relative humidity ≤60%<br>Tableting room / equipment: Room 3 / PKT-30<br>Temperature / humidity: 23.1 / 31%<br>Rail size (setting/actual): 8.5 mm<br>Feeder speed (10–50 RPM): 30<br>Tableting speed (20–60 RPM): 30

3. Results — Final tablet specifications

Item	Acceptance criterion	Result
Hardness (kgf)	≥3	3–4
Thickness (mm)	3.4–3.8	3.4–3.5
Weight (mg)	194 ± 3% (189–199)	195–196
Friability (%) (20 tablets, 25 RPM, 100 revolutions)	≤1.0%	Conforms
Disintegration (minutes)	≤50 minutes	Conforms

[시생산 기록서]

R&I			
담당	파트장	팀장	임원
결재			

번호 : 2024-146 담당자 : 이은정

고객사	레이델코리아	MKT담당	이윤제
제품명	위약	제품유형	
제형	제피정제	펀치	8파이 원형
성상		섭취방법	1일1회, 1회2정/병포장
목적	임상용	시생산일자	2024-12-17

* 성분배합비율

단위중량		200 mg	총량	3.0 kg		
**No.**	**원료 Code**	**원 료 명**	**배합비율**	**단위중량**	**투입량(g)**	**비 고**
1	100012308	유당	54.0303 %	108.0606 mg	1,620.9090	
2	100001586	결정셀룰로오스	41.1697 %	82.3394 mg	1,235.0910	
3	100000684	CMC칼슘/카르복시메틸셀룰로오스칼슘	1.0000 %	2.0000 mg	30.0000	
4	100001395	스테아린산마그네슘	0.8000 %	1.6000 mg	24.0000	
		[코팅]				
5	100000645	HPMC(히드록시프로필메틸셀룰로스)	2.2620 %	4.5240 mg	67.8600	
6	100001270	글리세린지방산에스테르	0.0750 %	0.1500 mg	2.2500	
7	100000761	이산화티타늄	0.1430 %	0.2860 mg	4.2900	
8	100000122	치자청색소	0.5200 %	1.0400 mg	15.6000	
9	100000563	발효주정	3.5500 %	7.1000 mg	106.5000	
		합 계	100.0000 %	200.0000 mg	3000.0000	

* 제조방법

No.	공 정 내 용	확인자
1	**1차 혼합**<br>이산화규소, 스테아린산마그네슘을 제외한 모든 원료를 20mesh에 체과하여 혼합한다.	
2	**2차 혼합**<br>위의 혼합물에 이산화규소, 스테아린산마그네슘을 20mesh에 체과하여 혼합한다.	
3	**타정**	
4	**코팅**	

*주의 사항 및 특이사항

Pilot Production Record

Record No.: 2024-146 Person in Charge: Lee Eun-jeong MKT Contact: Lee Yun-je

Field	Source entry / English rendering
Customer	Raydel Korea
Product name	Placebo
Product type	Not specified in source
Dosage form	Coated tablet
Punch	8 mm, round
Appearance	Blank in source
Directions for use	Once daily, 2 tablets per dose / bottle packaging
Purpose	For clinical trial use
Pilot production date	2024-12-17

Ingredient composition ratio

Unit weight: 200 mg Total batch: 3.0 kg

No.	Raw material code	Raw material name (English rendering)	Ratio	Unit weight	Input amount (g)	Remarks
1	100012308	Lactose	54.0303%	108.0606 mg	1,620.9090	
2	100001586	Crystalline cellulose	41.1697%	82.3394 mg	1,235.0910	
3	100000684	CMC calcium / calcium carboxymethylcellulose	1.0000%	2.0000 mg	30.0000	
4	100001395	Magnesium stearate	0.8000%	1.6000 mg	24.0000	
5	100000645	HPMC (hydroxypropyl methylcellulose)	2.2620%	4.5240 mg	67.8600	Coating
6	100001270	Glycerol fatty acid ester	0.0750%	0.1500 mg	2.2500	Coating
7	100000761	Titanium dioxide	0.1430%	0.2860 mg	4.2900	Coating
8	100000122	Gardenia blue color	0.5200%	1.0400 mg	15.6000	Coating
9	100000563	Fermented ethanol	3.5500%	7.1000 mg	106.5000	Coating
		Total	100.0000%	200.0000 mg	3000.0000	

Manufacturing method

No.	Step	Process description
1	First mixing	All raw materials except silicon dioxide and magnesium stearate are passed through a 20-mesh sieve and mixed.
2	Second mixing	Silicon dioxide and magnesium stearate are passed through a 20-mesh sieve and mixed with the mixture above.
3	Tableting	No further details entered in source.
4	Coating	No further details entered in source.

Precautions / special notes: blank in source.

* 제조내용 및 품질 체크사항

No.	공 정 내 용
1	혼합 [혼합실 : 19~25℃ / 60% 이하]<br>온 / 습도 : 20.5℃ / 27%<br>사별체 사이즈 : 20 mesh<br>[중량기록지]
2	타정 조건 [타정실 : 19~25℃ / 60% 이하]
3	결과<br>[최종 타정규격]

타정실 / 설비	3실/PKT-30
온 / 습도	23.1/31%
레일 사이즈 (셋팅/실제)	8.5mm
피더 속도 (10~50 RPM)	30
타정 속도 (20~60 RPM)	30

항 목	설정 기준	결 과
경도 (kgf)	3 이상	3-5
두께 (mm)	3.4~3.8	3.4-3.5
중량 (mg)	-	200-210
마손도 (%) (20T,25RPM,100회)	1.0% 이하	적합
붕해 (분)	50분 이하	적합

Manufacturing Details and Quality Check Items

No.	Item	Source entry / English rendering
1	Mixing	Mixing room: 19–25°C / relative humidity ≤60%<br>Temperature / humidity: 20.5°C / 27%<br>Sieve size: 20 mesh<br>[Weight record sheet] (blank area in source)
2	Tableting conditions	Tableting room: 19–25°C / relative humidity ≤60%<br>Tableting room / equipment: Room 3 / PKT-30<br>Temperature / humidity: 23.1 / 31%<br>Rail size (setting/actual): 8.5 mm<br>Feeder speed (10–50 RPM): 30<br>Tableting speed (20–60 RPM): 30

3. Results — Final tablet specifications

Item	Acceptance criterion	Result
Hardness (kgf)	≥3	3–5
Thickness (mm)	3.4–3.8	3.4–3.5
Weight (mg)	—	200–210
Friability (%) (20 tablets, 25 RPM, 100 revolutions)	≤1.0%	Conforms
Disintegration (minutes)	≤50 minutes	Conforms

Supplementary Table S4. Details of the plant registration form.

■ Form No. 8-2 Also available online: www.factoryon.go.kr

Plant Registration Certification Form

Reference No.	Date Received	Processing Time	Immediate

Company Information	Name of Company<br>COSMAXBIO, INC.	Tel.	
	Name of Chairperson<br>Moon Sung Ki	Date of Birth (Corporate Registration No.)<br>110111-0386022	
	Address of Chairperson (Address of Company)<br>30,Bio valley 3-ro,Jecheon-si,Chungcheongbuk-do		
Int raydel Address	Address of Plant<br>30,Bio valley 3-ro,Jecheon-si,Chungcheongbuk-do	Land Category<br>factory site	Property Status<br>Owned [v], Rented [ ]
	Plant Registration Date<br>013-11-18	Start Date of Business<br>1980-01-21	No. of Employees<br>Male: 124 Female: 60
	usiness Line (Classification Code)<br>anufacture of Health Foods (10797,21210)		
	Area of Site<br>19,131.08 ㎡	Area of Manufacturing Facilities<br>16,184.58 ㎡	Area of Other Facilities<br>7,951.47 ㎡
Registration Conditions			
Changes (Including Dates)			

I hereby apply for a Plant Registration Certificate in accordance with Article 12-3 of the Industrial Cluster Development and Factory Establishment Act.

2023 Year 03 Month 08 Day

Applicant 고하늘 (Signature or Stamp)

To Korea Industrial Complex Corporation

Required Documents	Not Required	Service Charge
		Fees stipulated by the local government (si, gun or gu) or responsible authority

I hereby certify that the above plant is registered in accordance with Article 16-1/16-2/16-3 of the Industrial Cluster Development and Factory Establishment Act.

2023 Year 03 Month 08 Day

Korea Industrial Complex Corporation

210mm×297mm[Paper 80g/㎡]
